# Psychological distress among individuals with a suicide attempt or suicidal ideation and suicide attempts patterns: first two years of the pandemic

**DOI:** 10.3389/fpsyt.2024.1366191

**Published:** 2024-03-08

**Authors:** Annekatrin Groh, Lydia Bahlmann, Lejla Colic, Alexandra Schulz, Ulrich W. Kastner, Udo Polzer, Martin Walter, Thomas Sobanski, Gerd Wagner

**Affiliations:** ^1^ Department of Psychiatry and Psychotherapy, Jena Center for Mental Health, Jena University Hospital, Jena, Germany; ^2^ Network for Suicide Prevention in Thuringia (NeST), Department of Psychiatry and Psychotherapy, Jena University Hospital, Jena, Germany; ^3^ German Center for Mental Health (DZPG), Partner Site Jena, Jena, Germany; ^4^ Department of Psychiatry, Psychotherapy, and Psychosomatic Medicine, Thueringen-Kliniken “Georgius Agricola” GmbH, Saalfeld, Germany; ^5^ Department of Psychiatry, Addiction, Psychotherapy and Psychosomatics, Klinikum am Europakanal, Erlangen, Germany; ^6^ Department of Psychiatry and Psychotherapy, Asklepios-Fachklinikum, Stadtroda, Germany; ^7^ Circuits Underlying Mental Health (C-I-R-C), Jena-Magdeburg-Halle, Germany

**Keywords:** suicide, suicide attempts, suicidal ideation, COVID-19, interrupted time series analysis, psychological stress, coping resources, mental health

## Abstract

**Background:**

The COVID-19 pandemic and related restrictions may have led to increased stress, particularly in people with mental health problems. Since stress factors play important role in the emergence of suicide attempts (SA) and suicidal ideation (SI), they may have been exacerbated by the pandemic, which could have led to an increased number of suicide attempts. Thus, we first investigated whether the pandemic affected personal stress experiences and appraisal of coping potential in individuals with and without SA and SI. In a second step, we analyzed the frequency and dynamics of SAs by patients admitted to a psychiatric university clinic over a period of four years.

**Methods:**

We examined stress experiences and appraisal of coping resources of inpatients recruited between March 2021 and February 2022 with SA (n=38), SI (n=27), and with mood disorder without SA or SI (n=45). In the second study, we investigated the time course of prospectively recorded patients with a suicide attempt (n=399) between January 1^st^ 2018 and December 31^st^ 2021 using interrupted time-series Poisson regression models.

**Results:**

There was a significant main effect of group (F[2,107]=6.58, p=0.002) regarding psychological stress levels, which was significantly higher in the SA and SI groups than in the psychiatric control group. No significant differences were found in the appraisal of coping resources or in the frequency of SAs before and during pandemic. However, the pandemic had a significant impact on the seasonal pattern of SAs.

**Conclusions:**

The pandemic increased psychological stress levels in individuals with SA and SI, which may be related to SI and do not necessarily result in SA. The pandemic did not affect the overall frequency of SA between March 2020 and December 2021, but interfered with the seasonal pattern of SA occurrence. Effective intervention strategies during a pandemic should include programs to strengthen the psychological resilience of people who are susceptible to mental health problems.

## Highlights

- Inpatients with suicide attempts (SA) and suicidal ideation (SI) reported similar levels of psychological distress.
- SAs and SIs reported higher psychological stress symptoms compared to psychiatric controls.
- No group differences were observed in the appraisal of coping resources.
- The frequency of SA during the pandemic did not differ from that before the pandemic.
- We found differences in the seasonality of SA during the pandemic compared to pre-pandemic.

## Introduction

On March 20^th^, 2020, the World Health Organization (WHO) declared the spread of the novel ‘Severe acute respiratory syndrome coronavirus 2’ (SARS-CoV-2) and the resulting coronavirus disease (COVID-19) a global pandemic [World Health Organization, (1)].

The pandemic and associated preventive measures increased stressors that have affected individuals and communities in different ways. One major source of stress has been uncertainty about the future, e.g., worries about individual health and about the well-being of relatives (2, 3). Social distancing measures and lockdowns (4) have led to increased loneliness and lack of social support for many people, in particular individuals with psychiatric disorders (5, 6). Furthermore, the pandemic has created additional challenges related to caregiving responsibilities (7). Thus, all of these factors have led to increased stress levels in many people around the world (8).

For example, several German cross-sectional studies with online surveys in the general population investigated the effects of pandemic-related restrictions on the extent of psychosocial stress and on mental health in the early phase of the pandemic and reported a significantly increased prevalence of psychological distress, e.g., from 39% before to 65.2% during the pandemic as well as of depressive and anxiety symptoms (6, 9, 10). Studies from other countries, e.g., the US, also reported an increase in psychological distress as a possible consequence of the pandemic itself and the preventive measures (11). It is assumed that the increase in stress experiences also leads in vulnerable people to an increase in mental health problems such as negative mood, hopelessness, anxiety, irritability or suicidal ideation (12). In a systematic review of data collected mainly in the early phase of the pandemic (between March and May 2020), Santomauro, Herrera (13) reported a 27.6% increase in the global prevalence of major depressive disorder and a 25.6% increase in anxiety disorders. As a consequence, psychiatrists and the media proclaimed at the onset of the pandemic that the global population would experience a “mental health tsunami” (14, 15). However, recent systematic reviews of a larger number of high-quality study data collected over a longer period of the pandemic showed that there was only a small increase in mental health symptoms in the general population (16–18). This means that the impact of the pandemic on mental health appears to be much more nuanced than was assumed at the beginning of the pandemic. It also appears that factors such as the appraisal of one’s own coping potential and the use of specific coping strategies have had a significant influence on mental health during the pandemic (19).

Notwithstanding, there were only few robust studies with vulnerable groups, such as people with pre-existing mental disorders or with previous suicide attempts, and it may be that some groups experience psychological distress and mental health issues differently from the general population. For example, in an Australian study individuals with mental disorders experienced higher levels of stress as a result of the pandemic than people without mental disorders (20). In that context, an especially vulnerable group in terms of experiencing stress may be individuals with a diathesis for suicidal behavior (SB) and/or suicidal ideation (SI). SB encompasses a spectrum of behaviors from suicide attempt to completed suicide. Mental disorders, in particular mood disorders, are considered a significant stress factor that significantly increases the risk of suicidal behavior (21). Previous studies reported a lifetime SA rate ranging from 19% to 50% for bipolar disorder (BD) and between 21% and 40% in MDD (22). A recent meta-analysis found a pooled estimate of suicide rate of 237.0 per 100,000 person-years for BD and 534.3 per 100,000 person-years for MDD, which is the highest suicide rate among mental disorders (23) and considerably higher than the global suicide rate of 9.0 per 100,000 inhabitants in 2019 (24).

Amplification of psychological stress due to pandemic-related factors such as social distancing, financial worries and increased worries about health and lack of adaptive coping have been discussed as possible triggers for SB, e.g., in people with mood disorders or substance use disorder (4, 25, 26). Similarly, it has also been shown that increased perceived stress during the pandemic was strongly correlated with the occurrence of suicidal ideation (27, 28). Thus, it is not clear if perceived stress, but also appraisal of coping skills during the pandemic is related to the occurrence of SB, or whether this applies more to the occurrence of SI. Even if previous epidemics (29) or socioeconomic crises (30) seems to have some influence on suicide rates, there is still too little knowledge about the factors that facilitate the transition from suicidal ideation to suicidal action (31).

Most studies on the potential triggers for SB during the Covid-19 pandemic were based on online surveys or meta-regression with extrapolation (13, 32, 33). A face-to-face interview with clinically well-characterized patients using standardized and comprehensive clinical assessment may therefore provide a more accurate evaluation of the possible interaction between SA and experienced stress during the pandemic.

One of the few studies based on structured face-to-face interview found that family problems such as marginalization, domestic violence and personal/health concerns such as fear or uncertainty were frequently cited as reasons for a suicide attempt during the Covid-19 pandemic (34). Tanaka and Okamoto (35) also described a simultaneous increase in the suicide rate and the number of calls about domestic violence in the first month of the pandemic. Following this line of reasoning, some authors have argued that the pandemic could lead to an increase in SB (36).

However, thus far, the studies on the occurrence of suicide attempts (SA) and suicides during the pandemic have been inconsistent. Studies from Germany analyzing suicide rates or the number of SA over several years have shown no significant increase in suicidal behavior in the first two years (37, 38) or even decreased number of SAs (39) in the first wave of the pandemic compared to the period before the pandemic. These results are supported by studies from Australia or Spain, for example (40–42). On the other hand, there are studies from Japan, India, Brazil, Czech Republic, and the United Kingdom that describe increased suicide rates (43–46), indicating country specific variability. In addition, in our recently published study (37), we found that the seasonal pattern of suicide attempts is significantly influenced by the pandemic.

Thus, in the present two-part mixed methods study we investigated perceived stress level, appraisal of coping skills during the pandemic and areas of life affected by the pandemic in individuals who have recently attempted suicide. We hypothesized that individuals with SA will exhibit higher level of stress and lower appraisal of coping skills due to pandemic-related factors compared to patients with a mood disorder and suicidal ideation as well as compared to patients with a mood disorder, but without SA/SI (Hypothesis #1).

In a second part of the study, we investigated the temporal course of the prospectively and systematically recorded number of suicide attempts from January 2018 to December 2021 from a large psychiatric university clinic in Jena, Germany. We hypothesized that, despite the negative results of some previous studies, the number of suicide attempts will increase during the COVID-19 pandemic and the dynamics of their occurrence will change (Hypothesis #2).

## Materials and methods

### Study #1 (hypothesis #1)

#### Participants

To investigate the first hypothesis, a sample of 110 adult inpatients with a recent SA or current SI or without SA/SI was recruited from March 2021 to February 2022 in the Departments of Psychiatry and Psychotherapy of Jena University Hospital and Thüringen-Kliniken “Georgius Agricola” in Saalfeld.

Inclusion criteria for the SAs were based on the Diagnostic and Statistical Manual of Mental Disorders (DSM)-5 criteria for the current suicide behavior disorder (SBD), which is one of eight conditions for further study included in Section III of the DSM-5 (47). The criteria explicitly define suicide attempt as “a self-initiated sequence of behaviors by an individual who, at the time of initiation, expected that the set of actions would lead to his or her death” (47, p. 801). Since mood disorders were the most frequently found mental disorders associated with suicidal behavior and suicidal ideation (23, 48, 49), we included only inpatients with or without suicidal ideation who also had a mood disorder. Thus, inclusion criteria for the inpatients with SI were presence of a mood disorder and current suicidal ideation but no current/past SBD. Inclusion criteria for the inpatients without SI/SA (psychiatric control group) were presence of a mood disorder, no current suicidal ideation and no current/past SBD. Exclusion criteria for all groups were acute psychosis, foreign language barriers, dementia, lack of compliance, and age <18 years. All participants consented in writing to participate in the study.

Thus, the sample included three groups of inpatients: individuals with a recent SA (n=38), individuals with a mood disorder and SI (n=27), and individuals with a mood disorder but without current SI and current or past SA (n=45). The majority of inpatients with SA (76%) have been diagnosed with a mood disorder (**Table 1**). Recruitment and data collection were conducted in parallel for all groups. For 5 individuals in the SA group, scores for the SI intensity were missing. The third psychiatric control group comprised individuals with a mood disorder and without current SI and current or past SBD. The local ethics committee of the Friedrich Schiller University, Jena, Germany, approved this part of the study.

**Table 1:** Sociodemographic and clinical data for the three investigated psychiatric groups.

Characteristic	Patients with SA (n= 38)	Patients with SI (n= 27)	Psychiatric control group (n= 45)
**Gender:** m/f/d (n [%])	11/26/1 [29%/68%/3%]	11/16/0 [41%/59%/0%]	19/26/0 [42%/58%/0%]
**Age:** Mean [SD]	32.7 [14.0]	34.2 [16.8]	48.9 [15.8]
**Education:** (n) 12-year school 10-year school 9-year school no graduation	11 22 4 1	14 9 4 0	20 23 2 0
**Family Status:** (n) married divorced single widowed in relationship	7 3 26 0 2	8 1 17 0 1	23 5 13 2 2
Mental disorder (ICD-10), n [%]: F 10.x F 31.3 F 32.x F 33.x F 60.x Intentional self-harm (ICD-10, X60-X84), n [%]: X60 X61 X62 X63 X64 X69 X70 X78 X81 X84	2 [5.4%] 1 [2.6%] 10 [26.3%] 18 [47.4%] 7 [18.4%] 4 [10.5%] 11 [28.9%] 5 [13.2%] 1 [2.6%] 3 [7.9%] 1 [2.6%] 4 [10.5%] 7 [18.4%] 1 [2.6%] 1 [2.6%]	0 [0%] 0 [0%] 11[40.7%] 16 [59.3] 0 [0%]	0 [0%] 0 [0%] 18 [40%] 27 [60%] 0 [0%]
**C-SSRS, suicide attempt:** (n) actual aborted interrupted	29 4 5		
**C-SSRS, intensity of suicide ideation last 3 months: mean [SD]**	3.9 [1.7]	3.0 [0.9]	/
	Mann-Whitney-U test: Z=3.2, p=0.001		
**SIS:** mean [SD]	11.71 [5.19]	/	/

The actual suicide attempt and the method used are shown as ICD-10 codes for intentional self-harm, X60-X84.

C-SSRS, Columbia Suicide Severity Rating Scale; ICD-10, International Statistical Classification of Diseases; SIS, Suicide Intent Scale; SA, suicide attempt; SD, standard deviation; SI, suicide ideation.

#### Assessments

Data collection involved a structured face-to-face interview, clinician- and patient-rated questionnaires, which were carried out by trained members of the study team (A.G., L.B., A.S.) under hygienic conditions. The interview included the assessment of sociodemographic data (age, gender, number of school years, family status), recording of previous and current SAs and the presence and intensity of SI, number of previous SAs, method and “intent to die” of the last SA, as well as the main diagnosis of mental disorder according to the criteria included in Chapter V(F) of the International Statistical Classification of Diseases (ICD-10). The average time between the SA and clinical interview was 31 days. The total duration of the clinical assessment was approximately 90 minutes, depending on the study participant. The data from the clinical interview are summarized in **Table 1**.

#### Intent to die

The intent to die, which is an important criterion for the definition of an SBD in DSM-5, was systematically evaluated in suicide attempters using the Pierce Suicide Intent Scale [SIS; (50)]. In this clinician-rated questionnaire, the objective circumstances (6 items) and the patient’s evaluation (4 items) related to the recent SA are recorded. In addition, there are two items that assess the potential lethality of SA. Each item is rated on an ordinal scale of 0, 1, or 2, with the total score ranging from 0 to 24. The higher the total score, the higher the patient’s intention to die. The questionnaire takes approx. 15 min.

#### Suicidal ideation and behavior

The presence of suicide attempts and suicidal ideation was assessed using the clinician-rated Columbia Suicide Severity Rating Scale [C-SSRS; (51)]. C-SSRS systematically asks a series of questions about suicidal thoughts, e.g., about the desire to be dead, thoughts about the suicide method or the existence of a specific plan and their intensity. In addition, C-SSRS scale systematically assess current and past SB encompassing actual attempts and following the definition of SBD in DSM-5, but also interrupted or aborted attempts [C-SSRS; (51)]. The questionnaire takes approx. 15 min.

#### Stress symptoms and reappraisal of coping skills

Symptoms of stress in the past 4 weeks related to pandemic, as explicitly stated in the instruction, were assessed by the Subclinical Stress Symptom Questionnaire [SSQ-25; (52)]. This self-report questionnaire comprises 25 items evaluating psychological and physical stress symptoms, ranging from 1 (not at all) to 5 (very intense). Psychological stress is measured by 15 questions about internal tension, nervousness, central issues and concerns (e.g., “I was easily irritated, annoyed or moody.”). Physical stress is assessed with 10 questions aimed at pain, weight changes, circulatory problems, insomnia (e.g., “I had trouble falling asleep, sleeping through or sleeping late “). SSQ-25 has good internal consistency and is suitable to differentiate different stress profiles even in samples with non-clinically significant mental health symptoms (52).

Individual appraisal of the threat posed by the pandemic and the associated measures as well as of the individual coping skills were assessed using the Primary Appraisal Secondary Appraisal (PASA) questionnaire (53). It is a 16-item self-report questionnaire specifically designed to measure cognitive appraisal processes in a stressful situation like Covid-19 pandemic (54). The two PASA subscales used in the present study measure primary appraisal, i.e., the person’s appraisal of the significance of the pandemic, e.g., as threatening, controllable or challenging and secondary appraisal, i.e., “self-concept of own competence” and “control expectancy”, which assesses coping resources available. Another PASA subscale used is the stress index, which is the difference between the values of the primary and secondary appraisal and provides a measure of stress perception. Higher score on the stress index indicates higher stress. Completing the SSQ-25 and PASA questionnaires takes about 5-10 minutes, respectively.

#### Areas of life affected by the pandemic

Individual stressful life events during the COVID-19 pandemic and their perceived stress were assessed using a structured interview in the following key life domains: Family/Partnership, Apartment, Social Life, Job, Finances, Leisure time, Health, Regeneration, and Life Perspective based on Satow (55). The patients were asked to indicate whether and, if so, which specific factors/burdens in the systematically surveyed life domains had led to an increased perception of stress during pandemic. With regard to life perspectives, for example, they were asked: “What factors have made you feel burdened in the area of life goals/life planning during the COVID-19 pandemic?” Subjects have to name these factors briefly and use the Likert scale from 1 (low) to 7 (high) provided to estimate the respective degree of stress. 11 persons of the SA group had missing values in the interview for the domains “Social Life” and “Regeneration”. This interview lasted about 20 minutes.

#### Statistical analysis

To investigate overall differences between the three groups in stress perceptions and appraisal of coping resources, analyses of variance (ANOVA) tests were done with the subscales of SSQ-25 and PASA questionnaires as dependent variables. To investigate the differences between the group means, *post-hoc* t-tests were performed, which were adjusted for multiple comparisons. For nominal variables, such as presence of stress in certain life domains, a Fisher’s exact test and for ordinal variables, a Kruskal-Wallis test were performed. Missing values were imputed with the overall mean values of the respective individual questions across all groups. Alpha threshold was set to 0.05.

### Study #2 (hypothesis #2)

#### Study design and participants

This study was part of a suicide prevention project (“Network for Suicide Prevention in Thuringia”), funded by the Federal Ministry of Health (BMG). We prospectively collected unique data on adults, 18 years or older, (n=399; mean age=41.88, standard deviation (SD)=19.36; 206 (51.63%) women) admitted after a suicide attempt to the Department of Psychiatry and Psychotherapy, Jena University Hospital. The study was conducted between January 1^th^, 2018 and December 31^th^, 2021. A suicide attempt was defined based on the DSM-5 criteria for the current SBD (47). Exclusion criteria were self-harm behavior in an altered mental state such as delirium or confusion, or if the act was ideologically motivated. The current diagnosis of SBD was also clearly delineated from the ‘non-suicidal self-injury.’ Based on the above criteria, 95 participants with SA were included in the study for 2018, 107 for 2019, 111 for 2020, and 86 for 2021. Six participants were excluded because the precise date of SA was not recorded. Prospective and systematic data collection within this project was additionally retrospectively checked for completeness via the hospital’s internal documentation system.

The government introduced significant measures in Germany and Thuringia State to contain the COVID-19 pandemic with the first restrictions starting in March 2020, strongly affecting social and public life. As a result, people reduced their private contacts substantially to a minimum. We used this time point to compare the frequency and the time course of SA before (Jan, 2018 to Feb, 2020; 220 suicide attempters included) and during the COVID-19 pandemic (Mar, 2020 to Dec, 2021; 179 suicide attempters included). The local ethics committee of the Friedrich Schiller University, Jena, Germany, also approved this part of the study.

#### Assessments

The data collection included prospectively collected sociodemographic data such as age and gender, the ICD-10 diagnosis of the concomitant psychiatric disorder, and SA-related information such as intent to die using SIS questionnaire, the date of the current suicide attempt, the current SA method, and the location of the SA.

#### Statistical analysis: modeling interrupted time-series

To model changes in SA frequency before and during the COVID-19 pandemic, we applied an interrupted time-series Poisson regression model using SPSS version 29.0 (https://www.ibm.com/de-de/analytics/spss-statistics-software). We modeled the impact of the pandemic, while controlling for the seasonal pattern and trend. We explored the interaction effects between the factor pandemic and seasonal patterns and trends. For modeling both covariates, i.e. seasonal pattern and trend, the time series of suicide attempts were decomposed using the additive model as implemented in the seasonal decomposition procedure in SPSS version 29.0 (https://www.ibm.com/docs/en/spss-statistics/29.0.0?topic=forecasting-seasonal-decomposition). The smoothed trend-cycle component was used as a covariate for the Poisson regression. This method was previously used for modeling the count time series (40, 56). The alpha threshold was 0.05.

## Results

### Study #1

#### Stress symptoms during the pandemic and the appraisal of coping resources

**Figure 1:**
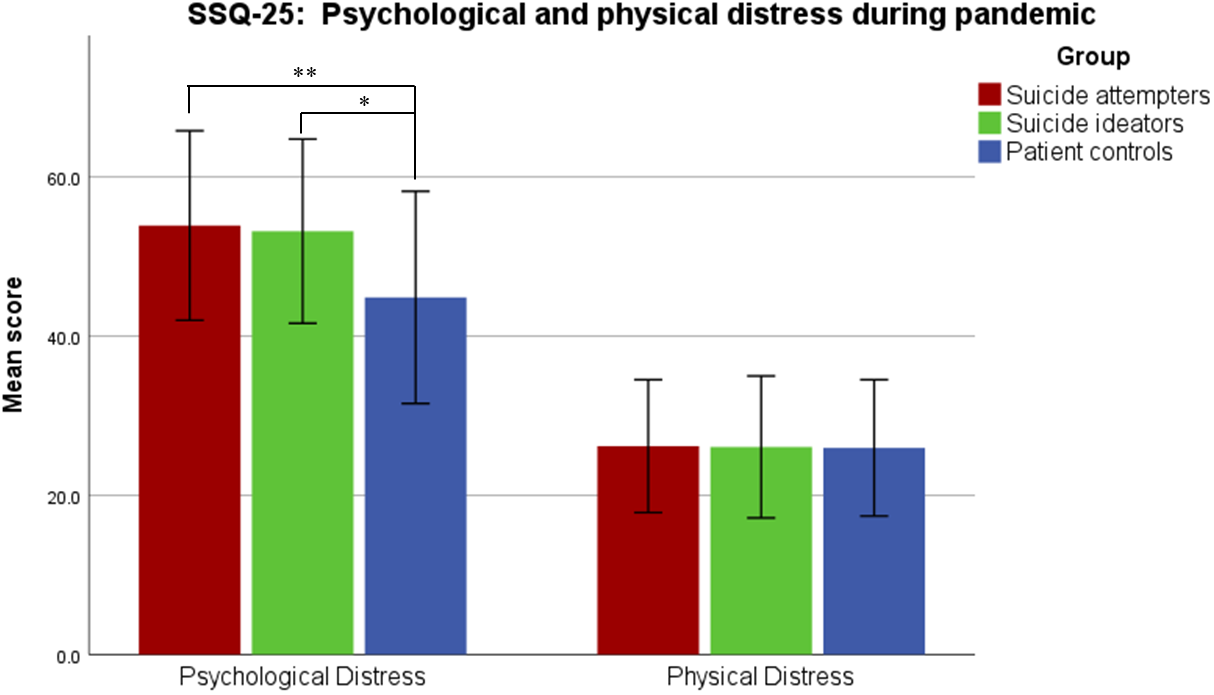
Psychological and physical stress symptoms experienced due to the COVID-19 pandemic, assessed with Subclinical Stress Symptom Questionnaire (SSQ-25). Error bars correspond to the first standard deviation. Patients with SA and SI reported significantly higher levels of psychological stress symptoms compared to the control patients. (*, p<0.05; **, p<0.01).

*SSQ-25*. As shown in **Table 2**, the ANOVA indicated an overall significant difference across three groups in the levels of psychological stress symptoms (F[2,107]=6.58, p=0.002; **Figure 1**), but not regarding levels of physical stress symptoms (F[2,107]=0.01, p=0.99; **Figure 1**). *Post-hoc* t-tests revealed that the groups with SA (t[81]=3.23, p=0.005, Bonferroni-adjusted) and SI (t[70]=2.69, p=0.02, Bonferroni-adjusted) had significantly higher level of psychological stress symptoms during the pandemic than the psychiatric control group (**Table 2**). Suicide attempts and suicidal ideation did not differ significantly from each other, as shown in **Table 2**.

**Table 2:** Reported stress and burden of the COVID-19 pandemic.

	Group with SA	Group with SI	Psychiatric control group	Statistical comparisons
SSQ-25				
Psychological stress symptoms^A^	53.9 [11.9]	53.2 [11.6]	44.9 [13.3]	F[2,107]=6.58, p=0.002 SAs vs PCs, p=0.005^D^ SIs vs PCs, p=0.02 ^D^ SAs vs SIs p>0.9
Physical stress symptoms^A^	26.2 [8.3]	26.1 [8.9]	26.0 [8.6]	F[2,107]=0.01, p=n.s.
PASA				
Primary appraisal subscale^A^	16.9 [4.4]	15.5 [5.1]	17.0 [3.6]	F[2,107]=1.18, p=n.s.
Secondary appraisal subscale^A^	14.7 [4.2]	15.4 [4.3]	16.0 [3.0]	F[2,107]=1.27, p=n.s.
Total stress index^A^	2.3 [7.2]	0.1 [8.1]	1.0 [5.5]	F[2,107]=0.84, p=n.s.
Individual burden of the pandemic				
“Family/Partnership” - frequency ^B^: - intensity ^C^:	n=29 [76.3%] 4.6 [1.4]	n=15 [55.6%] 4.3 [2.0]	n=24 [53.3%] 5.1 [1.5]	SA > PC, p=0.025 n.s.
“Apartment” - frequency ^B^: - intensity ^C^:	n=10 [26.3%] 4.2 [1.8]	n=10 [37.0%] 4.1 [2.0]	n=20 [44.4%] 4.8 [1.7]	n.s. n.s.
“Social Life” - frequency ^B^: - intensity ^C^:	n=23 [60.5%] 4.6 [1.6]	n=19 [70.4%] 5.5 [1.3]	n=43 [95.6%] 5.2 [1.3]	n.s. PC > SI, p=0.005
“Job” - frequency ^B^: - intensity ^C^:	n=22 [57.9%] 5.8 [1.4]	n=18 [66.7%] 5.4 [1.4]	n=27 [60.0%] 5.7 [1.4]	n.s. n.s.
“Finance” - frequency ^B^: - intensity ^C^:	n=10 [26.3%] 5.4 [1.6]	n=7 [25.9%] 4.4 [1.4]	n=12 [26.7%] 5.7 [1.8]	n.s. n.s.
“Leisure Time” - frequency ^B^: - intensity ^C^:	n=35 [92.1%] 5.7 [1.8]	n=23 [85.2%] 4.9 [1.5]	n=39 [86.7%] 5.2 [1.4]	n.s. n.s.
“Health” - frequency ^B^: - intensity ^C^:	n=23 [60.5%] 5.6 [1.4]	n=21 [77.8%] 5.2 [1.2]	n=35 [77.8%] 5.3 [1.6]	n.s. n.s.
“Regeneration” - frequency ^B^: - intensity ^C^:	n=14 [36.8%] 4.4 [1.6]	n=7 [25.9%] 4.3 [1.5]	n=22 [48.9%] 4.9 [1.7]	n.s. PC > SI, p=0.046
“Life Perspective” - frequency ^B^: - intensity ^C^:	n=18 [47.4%] 4.9 [1.4]	n=17 [63.0%] 4.9 [1.7]	n=36 [80.0%] 4.9 [1.8]	PC > SA, p=0.002 n.s.

^A^ Groups were analyzed using analysis of variance (ANOVA) test and post-hoc t-tests.

^B^ Groups were analyzed using Fischer’s exact test.

^C^ Groups were analyzed using Kruskal-Wallis-H test and post-hoc Mann-Whitney-U test.

^D^ Bonferroni-adjusted p-values for multiple post-hoc t-tests.

The data is presented as mean [SD]. PASA, Primary Appraisal Secondary Appraisal Questionnaire; SSQ-25, Subclinical Stress Symptom Questionnaire; SA, suicide attempt; SD, standard deviation; SI, suicide ideation; PC, patient controls; n.s., not significant.

*PASA*. There were no group differences regarding primary appraisal of the pandemic (F[2,107]=1.18, p=0.31), secondary appraisal of coping resources (F[2,107]=1.27, p=0.28), as well as the stress index (F[2,107]=0.84, p=0.43, **Table 2**). Thus, regarding the primary appraisal, groups with SA and SI did not evaluate the overall pandemic situation as significantly more threatening or challenging than a psychiatric control group. In addition, all groups rated the expectation of available coping skills similarly.

#### Areas of life affected by the COVID-19 pandemic

**Figure 2:**
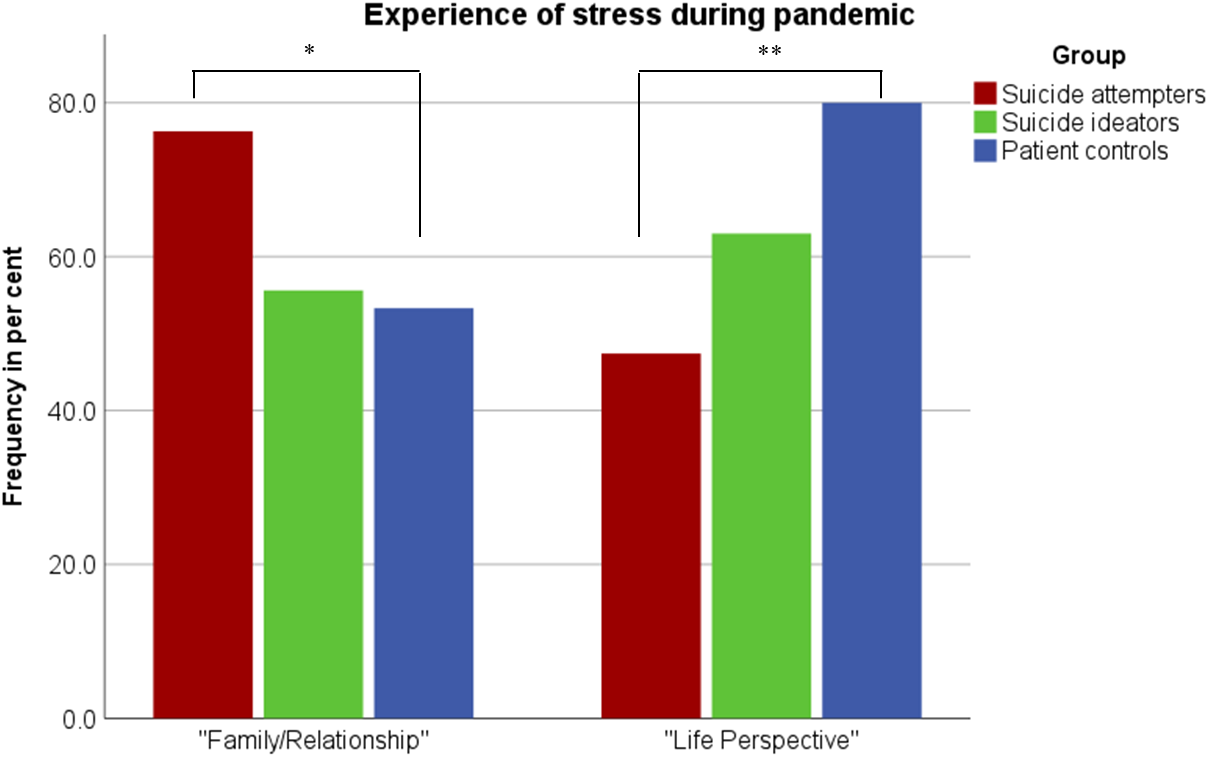
Frequencies of subjects reporting stress due to the COVID-19 pandemic in the domains of “family/partnership” and “life perspectives” (*, p<0.05; **, p<0.01).

The SA group was significantly more likely than the psychiatric control group to experience stress from the pandemic-related measures in the area “Family/Partnership” than psychiatric control group (Fischer’s exact test, p = .025) and on a trend level compared to the SI group (Fischer’s exact test, p = .068), see **Table 2** and **Figure 2**. Interestingly, patient controls felt more burdened by the pandemic than patients with SA in the area of “Life Perspectives/Life Planning” (Fischer’s exact test, p = .002) (**Table 2**, **Figure 2**). The SA group was also significantly more likely than the SI group to experience stress in the area “Regeneration” (Fischer’s exact test, p = .046). No significant differences were detected in other investigated life domains when comparing the SA group to both other groups. In addition, the psychiatric control group was significantly more likely to experience stress from the pandemic-related measures in the area “Social Life” (Fischer’s exact test, p = .005) and “Regeneration” (Fischer’s exact test, p = .046) than the SI group (**Table 2**).

The three groups did not show significant differences in the intensity of the experienced stress in the examined life domains, as the analysis with the Kruskal-Wallis-H test showed.

### Study #2

#### Effects of the pandemic on the frequency of suicide attempts

**Figure 3:**
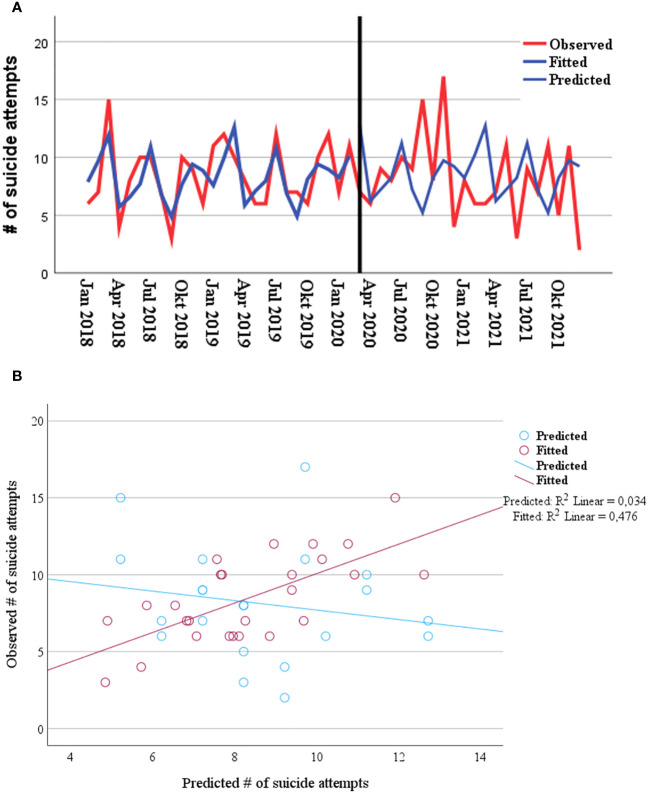
**(A)** Observed, fitted and predicted (during pandemic) values by applying simple seasonal model regarding the frequency of suicide attempts from 01 Jan 2018, until 31 Dec 2021. **(B)** Scatter plot and R-squared with observed data, fitted (R²=0.48) and predicted data (R²=0.03) for pre-pandemic (red dots) and pandemic (blue dots).

We did not observe a significant effect of the pandemic on the frequency of SA (Wald χ²=0.07, p=0.8). However, there was a significant interaction between the pandemic and the seasonal component (Wald χ²=15.14, p<0.001). The Goodness of Fit for the Pearson Chi-Square statistic resulted in the Value/df of 0.95 indicating no serious violation of the equidispersion. We additionally used time series modeler procedure in SPSS to predict the number of SA during the pandemic based on the pre-pandemic data. The simple seasonal model showed the best model fit of the pre-pandemic data. However, as illustrated in the **Figure 3**, this model did not have a good forecast (R²=0.03) for the actual numbers of SA during the pandemic, which supports the assumption of a changing seasonal pattern of SA occurrence.

## Discussion

Since the COVID-19 outbreak in March 2020, a variety of pandemic response actions took place. The contact minimization measures may have had a tremendous impact on people’s stress experiences, especially in individuals with mental disorders, which may consequently lead to an increase in suicidal ideation and suicide attempts. However, the differences between patients with SA and SI and patients without SA/SI in terms of perceived stress and appraisal of coping resources have not yet been investigated. In addition, previous findings on the possible increase in SA frequency during pandemic have been mixed. To answer these questions, in the first study, we investigated stress experiences and appraisal of personal coping skills via in-person structural clinical interviews and validated questionnaires across well-defined psychiatric groups. We, additionally, in the second study, examined the impact of the pandemic and associated measures on the number of suicide attempts by analyzing their time course from January 2018 to December 2021 in a large psychiatric university clinic in Germany.

We observed that both inpatients with SA and SI reported higher levels of psychological stress symptoms due to the pandemic than the psychiatric control group. Given that patients with SA also experience SI, the similarly high level of psychological stress symptoms would suggest that it is related to the development of suicidal thoughts rather than behavior. In support, population surveys indicate that psychological distress is a strong correlate of SI both in the pandemic (57, 58) as well as in the pre-pandemic period (59). In addition, current theories emphasize that clinical risk factors for suicidal ideation and suicidal acts partly overlap, but also have some unique features (60–63). Indeed, SI usually precedes SA, although a recent study reported that a larger number of individuals who had recently attempted suicide denied any suicidal thoughts (64). Nonetheless, given the marked differences in the prevalence of suicide ideation compared to action, most individuals with SI will not attempt suicide (65). In other words, it is relevant to consider suicidal ideation and attempted suicide as linked but also different phenotypes. This is also the main premise of current theories of suicidal behavior, e.g. the Three Step Theory (3ST) (66) that suicidal ideation is only one component of suicide risk and by itself is unlikely to lead to SB. The 3ST emphasizes that a combination of psychological pain and hopelessness as well as disrupted feeling of connectedness is the main cause for SI, but that transition from SI to SA is only possible if the capability to do so exists (67). Such a capability could be based on previous SA or non-suicidal self-harm.

Thus, the present findings suggest that although the COVID-19 pandemic and related restrictions might be a strong psychological stressor associated with an increase in suicidal ideation, other vulnerability factors such as cognitive, psychological or neurobiological vulnerability contribute to the transition from suicidal ideation to the manifest suicidal act. A converging body of evidence suggests specific differences in the so-called “suicide capability” among attempters compared to ideators (61).

In contrast to the SSQ-25 we did not find any significant differences in the PASA questionnaire assessing threats and challenges posed by the pandemic and the appraisal of personal coping resources. One suggested explanation for this result, which may seem contradictory at first glance, is that the questions of the SSQ-25 ask for stress symptoms in the last four weeks, whereas the PASA inquiries about symptoms and coping resources for the entire duration of the pandemic period. A recency effect can therefore not be ruled out in terms that more recent short-term experiences of the pandemic were described more challenging by the individuals with SI and SA, whereas the stress and coping level for the entire pandemic period were similarly described across groups. Furthermore, due to the cross-sectional nature of our study, we cannot discern if the groups experiencing SI would generally tend to experience and report higher levels of psychological distress also in the pre-pandemic time, i.e., in the sense that this association is unspecific to the pandemic context.

When asked systematically for stressful life events in various areas of life, individuals with SA were significantly more likely to experience stress in the area of “Family/Partnership” than the psychiatric control group and at the trend level than the SI group. There is some evidence from previous studies that pandemic-related lockdowns exacerbated existing difficulties in the relationship and familial cohabitation, leading to mental health difficulties and strained family relationships (68, 69). This last factor led to the onset or further exacerbation of domestic violence for some, especially those who were already at risk before (70). In our previous study we observed that by far most common motive given for a suicide attempt, especially for suicide re-attempts, was an interpersonal conflict (71). A longitudinal study, which investigated type and number of stressful life events in a 3-year follow-up period, observed that interpersonal conflicts, but also financial problems were the most robust predictors of a suicide attempt after adjusting for several demographic and clinical parameters (72). Also, the influential Interpersonal Psychological Theory of Suicide (IPTS) emphasizes the interpersonal problems in the emergence of suicidal desire (73). Our present finding also confirms the observation of the two previous studies, from India (34) and Japan (35), which reported an association between the increase in domestic violence and suicidal behavior during pandemic.

Thus, social disconnections and the intensification of existing inequalities in relationship quality might be one proximal factor responsible for the potential association between pandemic-related stay-at-home orders and suicidal behavior in vulnerable individuals, at least in the first waves of the pandemic. This finding further indicates that the amelioration of interpersonal relationships should be viewed as a key factor in suicide prevention - be it in its universal, selective or indicated form.

Surprisingly, the psychiatric control group with a mood disorder reported to be more affected by the pandemic regarding their life perspectives, such as future career plans or vacation trips. We do not know exactly why they perceived pandemic-related restrictions on life perspectives as particularly stressful. However, one reason might be that patients with SA and SI had already reduced life perspectives or life goals which was thus not affected by the pandemic to the same extent as it was in control patients, who did not develop suicidal ideations or showed suicidal behavior.

The other areas of life did not differ significantly between the groups. However, in almost all areas of life surveyed, with the exception of “finances”, most patients in all three groups reported pandemic-related burdens (**Table 2**). The overall stress level reported in the questionnaires was also relatively high, indicating that people with a mood disorder, regardless of the presence of SA/SI, were similarly affected by the pandemic and the associated restrictions (74, 75). Interestingly, all three groups reported relatively high level of appraisal of coping potential, which could be due at least in part to government assistance measures during pandemic, such as financial support.

Contrary to our hypothesis, there was no significant increase in suicide attempt numbers during the pandemic compared with the pre-pandemic time. This result is consistent with studies from high and upper-middle-income countries that also found no increase in the number of suicide attempts and deaths by suicide during the pandemic (37, 42, 76). There are a number of reasons for this lack of increase in SA frequency. The widely reported increases in psychological distress and mental health problems may have indeed increased suicidal ideation, which in turn does not necessarily lead to increases in suicide attempts (21, 61). In support, previous studies have described the association between acute stress and anxiety and an increase in suicidal ideation in the general population (32, 77) but not suicide attempts. Suicidal behavior is related not only to proximal factors, such as increased experience of stress, but also to predisposing factors, such as genetic and/or neurocognitive factors (21, 31). Together with the results of the first part of our study, we may therefore speculate that the psychological distress would not directly lead to SA unless there is an already present high vulnerability, e.g., poor decision-making (78). Lastly, some studies report stable levels not only for SA but also for SI during the pandemic (75). However, it cannot be ruled out that the varying strength of the restriction measures, the different levels of state support and the cultural differences in the individual countries have a specific negative but also protective influence on people at risk of suicidal behavior (79). Future global efforts could help to identify the systemic protective factors.

In our analysis, we did observe a significant interaction between the pandemic and seasonal components, where the seasonality of the SA trends was interrupted. Before the pandemic, the highest frequency of suicide attempts was in spring and late summer. During the pandemic, on the other hand, most suicide attempts occurred in fall 2020, with the start of the second wave of the pandemic. Our previous study (37) in the same federal state (Thuringia), but in a rural area, also showed similar frequencies of suicide attempts before and during the pandemic, but significant changes in seasonality. Previous studies have shown that there are seasonal clusters in the occurrence of suicides (80, 81), but these are heterogeneous in form, amplitude, as well as in the seasonal patterns of specific subgroups and between countries (82). We speculate that the observed changes in the seasonal variation in SA may be related to limited access to suicide means due to pandemic-related measures, such as stay-at-home orders, and/or due to changes in outdoor/indoor spent time [i.e., reported effect of daily sunshine on suicide frequency (83)] and/or pandemic-related changes in personal relationships, employment status and media reporting (84). Future prospective studies should collect corroborating data for activity patterns of individuals vulnerable to SA.

To sum up, SB is a complex and multifaceted phenomenon that encompasses biological, psychological and environmental factors, which interact in such an unfavorable way that a person decides to end their life. Although the pandemic has brought with it all the putative risk factors to trigger suicidal behavior, we have not seen an increase in SA numbers over a period of almost two years of the pandemic. This means that individual risk factors that constitute “suicidal capability” are necessary for suicidal thoughts to be acted upon. Further studies in subjects with SA are therefore needed to better understand this capability.

## Limitations

Participation in the face-to-face interview was voluntary, making it more likely that individuals who were open about their SA or SI and experienced distress were preferentially included in the study. Individuals who did not choose to participate may thus have exhibited higher levels of distress. Moreover, the study was conducted cross-sectionally over the second to fifth wave of the pandemic, which took place from the end of September 2020 to February 2022 (85). These waves differed in their severity of contact measures and it may be that various waves were perceived as differently “dangerous.” Thus, the recorded stress levels may have differed between participants due to the different survey periods. Regarding the time series analysis, it seems conceivable that individuals received initial care in an emergency department after a suicide attempt without being referred to psychiatry for further treatment. However, this would be in contrast to the situation before the pandemic began, where it was common for almost every patient to be referred to a psychiatric clinic for further evaluation of the suicide risk. In addition, 5 subjects in the SA group had missing values for the intensity of suicidal thoughts.

## Conclusion

In summary, we found increased pandemic-related psychological stress levels in vulnerable groups of inpatients with suicide attempt and suicidal ideation compared to a psychiatric control group, but no differences in the appraisal of coping resources. We also found that suicide attempters were more affected by the pandemic in the area of family and partnerships than the other two patient groups. These effects should be carefully investigated in further studies in order to draw final conclusions. Furthermore, the Covid-19 pandemic and its restriction measures have no direct effect on the SA frequency overall, but on the seasonal fluctuations. However, the long-term consequences of the pandemic and associated measures could have an impact on suicidal behavior in the future, as shown by a trend reversal in suicide rates in 2022 among older people in a recent study (86). It is therefore imperative to set up real-time and reliable monitoring of suicides and attempted suicides in order to implement targeted and timely measures and thus accelerate suicide prevention.

Finally, given the significance of suicidal behavior, there is undoubtedly an urgent need for the further development of effective psychosocial/psychotherapeutic therapies that have a specific focus on the prevention of suicidal behavior and on dealing with suicidal ideation. In our recent systematic review, such interventions were shown to be effective in preventing suicide re-attempts and suicide (87).

## Data availability statement

The raw data supporting the conclusions of this article will be made available by the authors, without undue reservation.

## Ethics statement

The studies involving humans were approved by ethics committee of the Friedrich Schiller University, Jena, Germany. The studies were conducted in accordance with the local legislation and institutional requirements. The participants provided their written informed consent to participate in this study.

## Author contributions

AG: Data curation, Formal Analysis, Investigation, Writing – original draft, Writing – review and editing. LB: Data curation, Investigation, Writing – original draft, Writing – review and editing. LC: Writing – original draft, Writing – review and editing. AS: Investigation, Writing – original draft, Writing – review and editing. UK: Writing – original draft, Writing – review and editing. UP: Writing – original draft, Writing – review and editing. MW: Supervision, Writing – original draft, Writing – review and editing. TS: Project administration, Supervision, Writing – original draft, Writing – review and editing. GW: Conceptualization, Data curation, Formal Analysis, Funding acquisition, Investigation, Supervision, Writing – original draft.
